# *Tet2* loss leads to hypermutagenicity in haematopoietic stem/progenitor cells

**DOI:** 10.1038/ncomms15102

**Published:** 2017-04-25

**Authors:** Feng Pan, Thomas S. Wingo, Zhigang Zhao, Rui Gao, Hideki Makishima, Guangbo Qu, Li Lin, Miao Yu, Janice R. Ortega, Jiapeng Wang, Aziz Nazha, Li Chen, Bing Yao, Can Liu, Shi Chen, Ophelia Weeks, Hongyu Ni, Brittany Lynn Phillips, Suming Huang, Jianlong Wang, Chuan He, Guo-Min Li, Tomas Radivoyevitch, Iannis Aifantis, Jaroslaw P. Maciejewski, Feng-Chun Yang, Peng Jin, Mingjiang Xu

**Affiliations:** 1 Department of Biochemistry and Molecular Biology, Sylvester Comprehensive Cancer Center, University of Miami Miller School of Medicine, 1011 NW 15th Street, Room 411, Gautier Building, MC R629, Miami, Florida 33136, USA; 2 Department of Pediatrics, Herman B. Wells Center for Pediatric Research, Indiana University School of Medicine, Indianapolis, Indiana 46202, USA; 3 Department of Biological Sciences, Florida International University, Miami, Florida 33199, USA; 4 Departments of Human Genetics, Emory University School of Medicine, Atlanta, Georgia 30307, USA; 5 Neurology, Emory University School of Medicine, Atlanta, Georgia 30307, USA; 6 Division of Neurology, Department of Veterans Affairs Medical Center, Atlanta, Georgia 30033, USA; 7 Department of Hematology and Oncology, Tianjin Medical University Cancer Institute and Hospital, National Clinical Research Center for Cancer, Key Laboratory of Cancer Prevention and Therapy, Tianjin 300060, China; 8 Department of Translational Hematology and Oncology Research, Taussig Cancer Institute, Cleveland Clinic, Cleveland, Ohio 44195, USA; 9 Department of Chemistry and Institute for Biophysical Dynamics, University of Chicago, Chicago, Illinois 60637, USA; 10 Department of Biochemistry and Molecular Biology, Norris Comprehensive Cancer Center, University of Southern California Keck School of Medicine, Los Angeles, California 90033, USA; 11 Department of Pathology, University of Illinois at Chicago, Chicago, Illinois 60607, USA; 12 Department of Biochemistry and Pharmacology, Emory University School of Medicine, Atlanta, Georgia 30307, USA; 13 Department of Biochemistry and Molecular Biology, University of Florida, Gainesville, Florida 32611, USA; 14 Department of Developmental and Regenerative Biology, Black Family Stem Cell Institute, Icahn School of Medicine at Mount Sinai, New York, New York 10029, USA; 15 Howard Hughes Medical Institute and Department of Pathology, NYU School of Medicine, New York, New York 10016, USA; 16 NYU Cancer Institute and Helen L. and Martin S. Kimmel Center for Stem Cell Biology, NYU School of Medicine, New York, New York 10016, USA

## Abstract

TET2 is a dioxygenase that catalyses multiple steps of 5-methylcytosine oxidation. Although *TET2* mutations frequently occur in various types of haematological malignancies, the mechanism by which they increase risk for these cancers remains poorly understood. Here we show that *Tet2*^−/−^ mice develop spontaneous myeloid, T- and B-cell malignancies after long latencies. Exome sequencing of *Tet2*^−/−^ tumours reveals accumulation of numerous mutations, including *Apc*, *Nf1*, *Flt3*, *Cbl*, *Notch1* and *Mll2*, which are recurrently deleted/mutated in human haematological malignancies. Single-cell-targeted sequencing of wild-type and premalignant *Tet2*^−/−^ Lin^−^c-Kit^+^ cells shows higher mutation frequencies in *Tet2*^−/−^ cells. We further show that the increased mutational burden is particularly high at genomic sites that gained 5-hydroxymethylcytosine, where TET2 normally binds. Furthermore, *TET2*-mutated myeloid malignancy patients have significantly more mutational events than patients with wild-type *TET2*. Thus, *Tet2* loss leads to hypermutagenicity in haematopoietic stem/progenitor cells, suggesting a novel *TET2* loss-mediated mechanism of haematological malignancy pathogenesis.

## Abstract (web summary)

TET2 catalyses DNA demethylation and is mutated in various blood cancers; in particular *Tet2* null mice develop haematological neoplasms. Here the authors show that this effect could be due to the increased frequency of mutation associated with TET2 loss in haematopoietic stem/progenitor cells.

Ten eleven translocation methylcytosine dioxygenases (TET1/2/3) catalyse the conversion of 5-methylcytosine (5mC) to 5-hydroxymethylcytosine (5hmC) and can further oxidize 5hmC to 5-formylcytosine (5fC) and 5-carboxylcytosine (5caC)^1^,^2^,^3^. 5fC and 5caC can then be removed by thymine DNA glycosylase (TDG) of base excision repair (BER)^4^. Alternatively, deamination may occur at 5hmC sites by AID/APOBEC cytidine deaminases to generate 5-hydroxymethyluracil (5hmU), which can also be repaired by BER^5^. Therefore, DNA methylation and TETs/TDG-BER-driven DNA demethylation form a complete cycle of dynamic cytosine modifications. The oxidation and demethylation of 5mC in the genome are regulated in a sophisticated manner. Genetic inactivation of *Tdg* and *Tets* leads to prominent alterations of CpG modifications at various gene regulatory regions. This raises the possibility that TETs/TDG-BER-mediated cytosine modifications may be widespread across the whole genome.

*TET2* is one of the most commonly mutated/deleted genes in adult myeloid malignancies, including ∼30% of cases of myelodysplastic syndrome (MDS), 20% of myeloproliferative neoplasms (MPNs), 17% of *de novo* acute myeloid leukaemias (AMLs), 30% of secondary AMLs and 50–60% of chronic myelomonocytic leukaemias^6^,^7^,^8^,^9^. Somatic *TET2* mutations also occur in T-cell lymphomas (such as angioimmunoblastic T lymphomas, 33%)^10^ and B-cell non-Hodgkin lymphomas (diffuse large B-cell lymphoma, 12%; mantle cell lymphoma, 4%)^11^,^12^. Mutations in *TET2* are also prevalent in healthy individuals over 70 years of age (>5%) and are often associated with clonal haematopoiesis^13^. These results indicate that *TET2* mutations are ancestral events that drive nonmalignant clonal outgrowth and facilitate haematological malignancy transformation. Indeed, *Tet2* loss in mice leads to increased haematopoietic stem cell (HSC) self-renewal and subsequent development of myeloid malignancies^14^,^15^,^16^,^17^. Loss-of-function *TET2* mutations and *TET2* loss result in aberrant 5mC and 5hmC profiles^14^,^18^, and we recently showed that TET2 likely requires its catalytic activity in HSC/haematopoietic progenitor cells (HPCs) to exert a tumour-suppressive function^19^. However, the mechanisms by which *TET2* loss leads to diverse haematological malignancies remain largely unknown.

Accumulations of mutations in HSCs/HPCs can be deleterious to haematopoietic function and promote haematological malignancy. Here we find, using our *Tet2*^−/−^ mouse models and combined biological, bioinformatics and genetic approaches, that TET2 safeguards HSCs/HPCs against genomic mutagenicity. Exome sequencing of *Tet2*^−/−^ tumours and targeted single-cell exome sequencing of premalignant wild-type (WT) and *Tet2*^−/−^ HSCs/HPCs show that *TET2* loss leads to genomic hypermutability in HSCs/HPCs. We further see that *Tet2* loss leads to a significantly higher mutational frequency at genomic sites that gained 5hmC on *Tet2* loss, where TET2 normally binds. Our results indicate that TET2 and TET2-mediated 5 mC oxidation safeguard cells against genomic mutagenicity. These findings suggest a novel mechanism contributing to *TET2* loss-mediated pathogenesis in a diverse array of haematological malignancies.

## Results

### *Tet2* ^−/−^ mice develop myeloid and lymphoid malignancies

**Figure 1:**
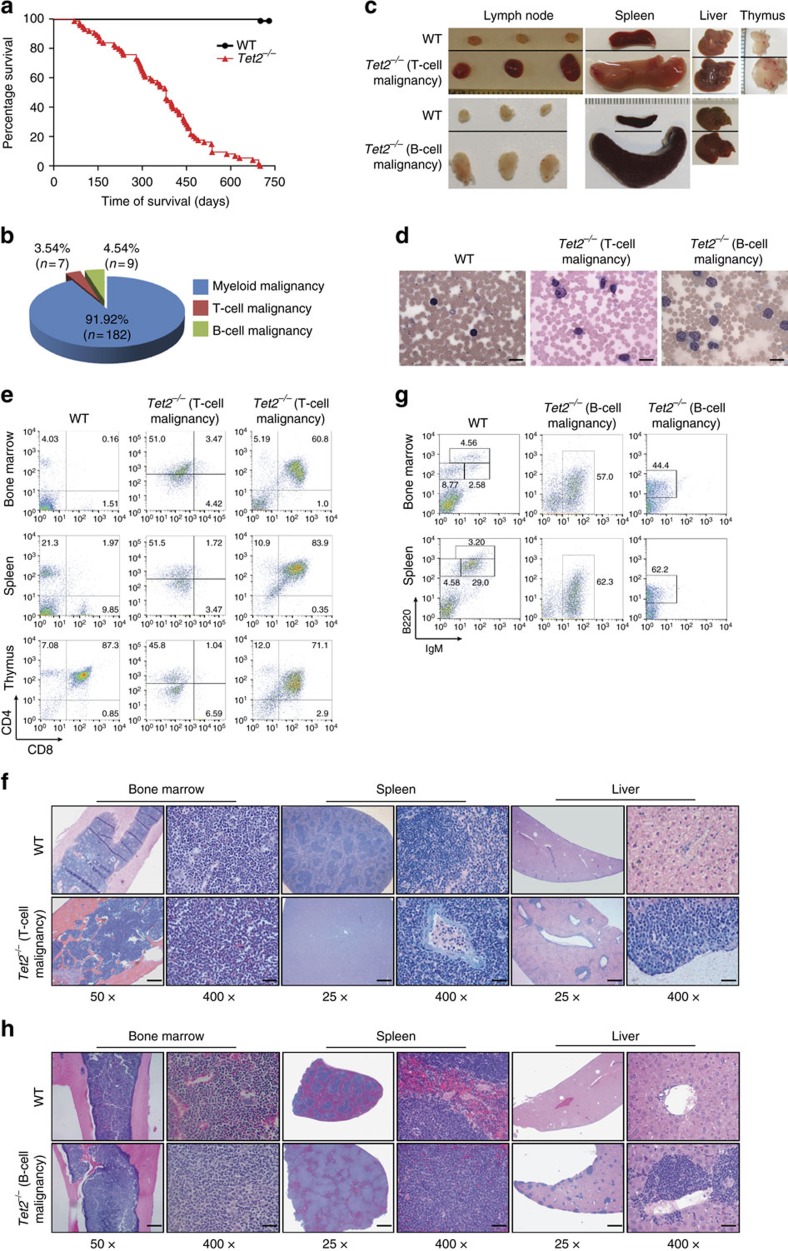
*Tet2*^−/−^ mice develop lethal lymphoid malignancies involving both T- and B-cell lineages. (**a**) Survival of WT (*n*=67) and *Tet2*^−/−^ (*n*=198) mice over time. (**b**) Proportions of haematological malignancy types developed in 198 *Tet2*^−/−^ mice. (**c**) Gross morphologies of lymph nodes, spleen, liver and/or thymus from representative *Tet2*^−/−^ mice with T-cell (upper) or B-cell (lower) malignancy and age-matched WT mice. (**d**) May–Grünwald–Giemsa-stained PB smears prepared from a representative WT mouse and *Tet2*^−/−^ mice with T- and B-cell malignancies (scale bar, 20 μm). (**e**) Flow cytometric analysis of the T-cell lineage (CD4/CD8) in BM, spleen and thymus of representative *Tet2*^−/−^ mice with T-cell malignancy and an age-matched WT mouse. (**f**) Histological analysis of H&E-stained sections of femur, spleen and liver from a representative *Tet2*^−/−^ mouse with T-cell malignancy and an age-matched WT mouse (scale bar, × 25, 200 μm; × 50, 100 μm; × 400, 12.5 μm). (**g**) Flow cytometric analysis of the B-cell lineage (B220/IgM) in BM and spleen of representative *Tet2*^−/−^ mice with B-cell malignancy and an age-matched WT mouse. (**h**) Histological analysis of H&E-stained sections of femur, spleen and liver from a representative *Tet2*^−/−^ mouse with B-cell malignancy and an age-matched WT mouse (scale bar, × 25, 200 μm; × 50, 100 μm; × 400, 12.5 μm).

To determine the complete spectrum of haematological malignancies caused by *Tet2* loss *in vivo*, we conducted a 2-year follow-up study on a cohort of 198 *Tet2*^−/−^ and 67 WT mice. All *Tet2*^−/−^ mice developed spontaneous lethal haematological malignancies with survival durations ranging from 3 to 22 months, whereas no abnormalities were detected in the haematopoietic organs of WT mice (Fig. 1a,b). Consistent with previous observations^14^,^15^,^16^, 92% of these *Tet2*^−/−^ mice developed myeloid malignancies, as indicated by monocytosis/neutrophilia, hepatosplenomegaly and marked expansion of well-differentiated myeloid cells or erythroid precursors in the bone marrow (BM), spleen and liver (Supplementary Fig. 1). Analyses of these 198 *Tet2*^−/−^ mice also showed that 3.5% of *Tet2*^−/−^ mice developed T-cell malignancies and 4.5% of *Tet2*^−/−^ mice developed B-cell malignancies (Fig. 1b–h, Supplementary Fig. 2 and Supplementary Tables 1 and 2). These mice displayed marked lymphocytosis consisting of atypical lymphocytes, lymphadenopathy, hepatosplenomegaly and enlarged thymuses, in most cases with T-cell malignancies (Fig. 1c,d).

Flow cytometric analyses of the spleen and BM cells from seven *Tet2*^−/−^ mice with T-cell malignancies revealed dominant proportions of CD3^+^ T lymphocytes with a high forward scatter; five of these seven animals aberrantly expressed CD4 in their T lymphocytes that were mostly positive for CD44 and PD1 (Fig. 1e, Supplementary Fig. 2a,b and Supplementary Table 1). BM, spleen, liver and thymus had atypical lymphoid infiltrates effacing or distorting the normal architecture of these organs (Fig. 1f and Supplementary Fig. 2c). Atypical lymphocytes were medium sized, with a smaller amount of cytoplasm and irregular nuclei relative to WT. The spleen showed diffuse lymphoid infiltrates involving both red and white pulp. The liver demonstrated sinusoidal and perivascular infiltration. In addition, splenic CD3^+^ cells from each of the tested *Tet2*^−/−^ mice with monomorphic T-cell infiltrations showed clonal T-cell receptor rearrangement patterns (Supplementary Fig. 2f).

In nine *Tet2*^−/−^ mice with B-cell malignancies, spleen and BM cells were dominantly B220^+/lo^IgM^+/lo^CD19^+^CD43^+^ TdT^−^ B lymphocytes with a high forward scatter (Fig. 1g, Supplementary Fig. 2d and Supplementary Table 2). B lymphocytes from four of these mice expressed CD5 (Supplementary Table 2). Neoplastic monomorphic expansions of B cells were thus observed in these *Tet2*^−/−^ mice. Atypical lymphoid infiltrates were identified in BM, spleen, liver, lymph node and small intestine (Fig. 1h and Supplementary Fig. 2e). Atypical lymphocytes were predominantly medium sized, with a smaller amount of cytoplasm relative to WT, and with irregular nuclear vesicular and condensed nuclear chromatin. The spleen showed effacement of normal architecture with nodular lymphoid infiltrate involving predominantly white pulp. The liver displayed perivascular and sinusoidal infiltration. The intestine showed lymphoepithelial lesions with lymphoid nodules. In addition, splenic B220^+^ cells from each of the tested *Tet2*^−/−^ mice with monomorphic B-cell infiltrations were clonal for IgH D–J rearrangement (Supplementary Fig. 2g).

### Lymphoid malignancies in *Tet2* ^−/−^ mice are transplantable

**Figure 2:**
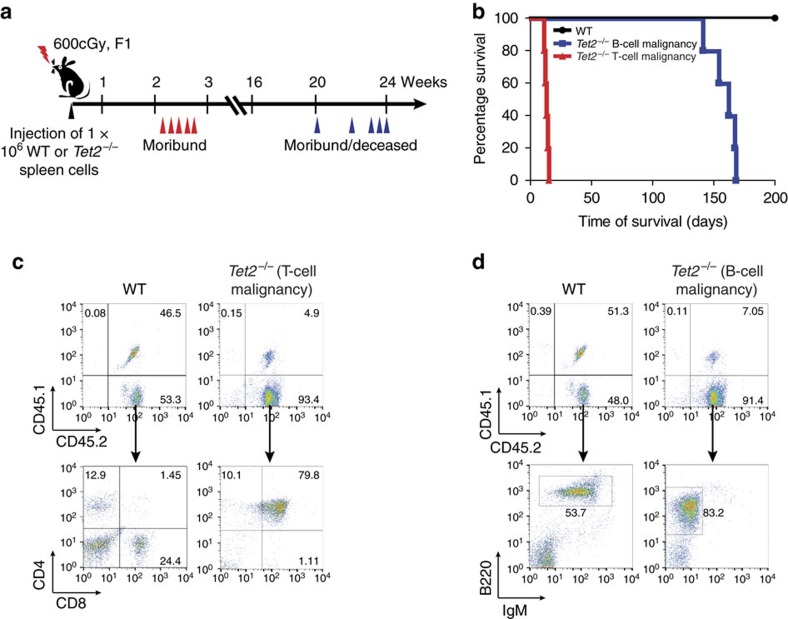
T- and B-cell malignancies in *Tet2*^−/−^ mice are transplantable to sublethally irradiated secondary WT recipients. (**a**) Tumour transfer schema. Spleen cells (1 × 10^6^) from representative *Tet2*^−/−^ mice with a T- or B-cell malignancy or from an age-matched WT mouse were injected into sublethally irradiated (600 cGy) CD45.1^+^/CD45.2^+^ F1 recipients. (**b**) Kaplan–Meier survival curve of sublethally irradiated recipients (five mice/donor) transplanted with spleen cells from *Tet2*^−/−^ mice with T-cell (red) or B-cell (blue) malignancies or from one WT (black) mouse. (**c**) Flow cytometric analyses of PB T-cell lineage (CD4/CD8) donor cells (CD45.2^+^) from a mouse receiving spleen cells from a WT mouse or a *Tet2*^−/−^ mouse with T-cell malignancy. (**d**) Flow cytometric analyses of peripheral blood B-cell lineage (B220/IgM) donor cells (CD45.2^+^) from a mouse receiving spleen cells from a WT mouse or a *Tet2*^−/−^ mouse with B-cell malignancy.

To evaluate the malignant nature of the abnormally infiltrated T and B lymphocytes in *Tet2*^−/−^ mice, spleen cells from one WT mouse and two *Tet2*^−/−^ mice with malignancies, one with a T-cell clone (G3-6) and one with a B-cell clone (G3-185), were transplanted into sublethally irradiated WT recipients (Fig. 2a). No recipient receiving WT spleen cells developed any evidence of disease within 6 months of transplantation (Fig. 2b). In contrast, all mice receiving spleen cells from *Tet2*^−/−^ mice with T- or B-cell malignancy developed diseases with characteristics similar to those observed in primary mouse, for example, elevated WBC counts, lymphocytosis, splenomegaly, enlarged lymph nodes and premature death (Fig. 2b). Flow cytometric analysis of peripheral blood (PB) cells of the recipients revealed infiltration of uniform, donor cell-derived, T- (CD45.2^+^CD4^+^CD8^+^) or B- (CD45.2^+^B220^+^IgM^low^) cell populations, similar to what we saw in the respective primary *Tet2*^−/−^ mouse (Fig. 2c,d). As *Tet2*^−/−^ B- and T-cell malignancies were transplantable into sublethally irradiated WT mice, infiltrating T or B lymphocytes in these *Tet2*^−/−^ mice are indeed malignant/neoplastic. Collectively, these data demonstrate that, in addition to myeloid malignancies, *Tet2*^−/−^ mice develop lethal T- and B-cell malignancies with features most closely resembling human peripheral T-cell lymphoma not otherwise specified and acute B-lymphocytic leukaemia, respectively. These findings are consistent with clinical observations that loss-of-function mutations in *TET2* are frequent in both myeloid and subtypes of B- and T-cell malignancies^6^,^7^,^8^,^9^,^10^,^11^,^16^.

### *Tet2* loss leads to hypermutagenicity in HSCs/HPCs

**Figure 3:**
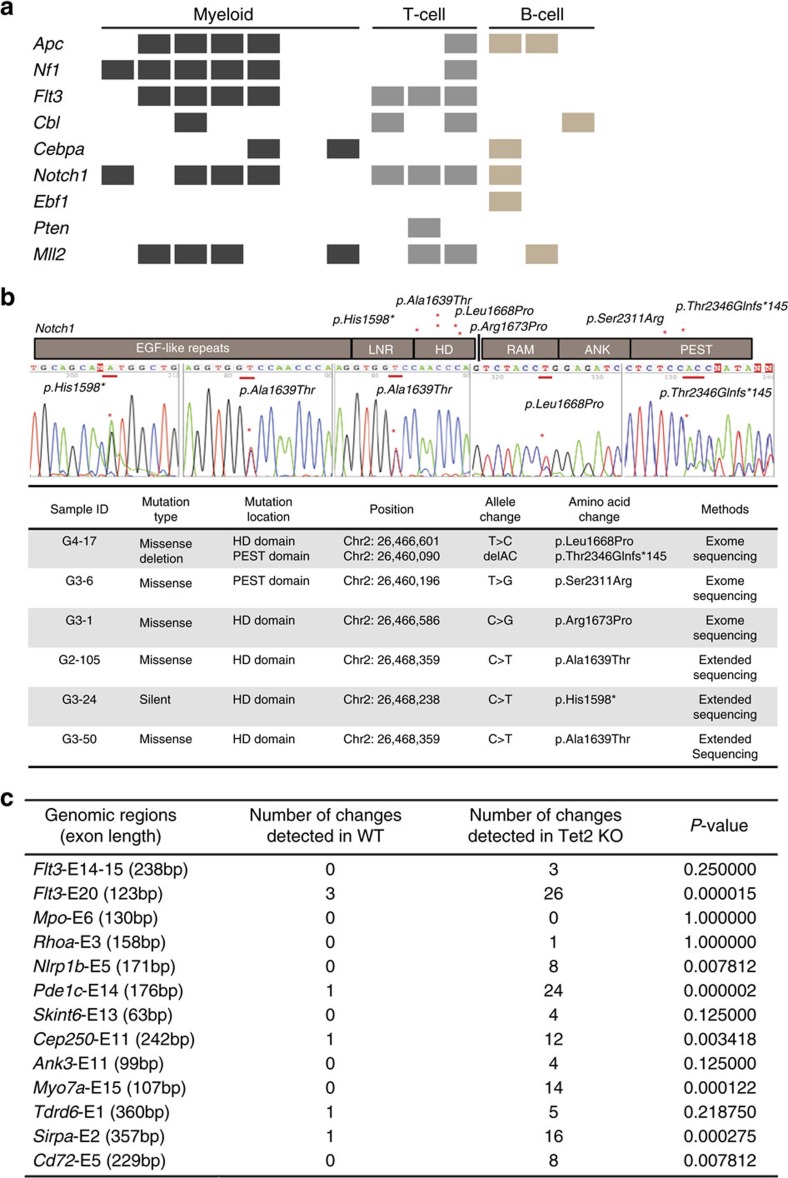
WES reveals mutations in TET2-deficient tumours. (**a**) Selected recurrent gene mutations in *Tet2*^−/−^ tumours of myeloid, B- or T-cell lineage as identified by WES. (**b**) *Notch1* gene mutations identified by exome-sequencing and/or Sanger sequencing in six *Tet2*^−/−^ T-cell tumours are summarized (lower). Sanger sequence traces of four *Tet2*^−/−^ T-cell tumours with heterozygous mutations in *Notch1* are shown (middle). The mutational locations are shown as red asterisks in the mouse NOTCH1 protein schematic representation (upper). (**c**) Significantly more mutations are found in premalignant *Tet2*^−/−^ LK cells compared to WT LK cells by single-cell-targeted exome sequencing (Fisher's exact test, *P*<0.001).

The kinetics and the involvement of multiple lineages by haematological malignancies in *Tet2*^−/−^ mice suggest that additional genetic lesions may be acquired in these mice, resulting in the pathogenesis and/or progression of various haematological malignancies. To explore this possibility, comparative genomic hybridization arrays and whole-exome sequencing (WES) were performed using tumour and non-tumour cells from *Tet2*^−/−^ mice with myeloid, T- or B-cell malignancies. Comparative genomic hybridization arrays identified a variety of structural chromosomal abnormalities in myeloid, T- and B-cell malignancies (Supplementary Fig. 3a and Supplementary Data 1). Further examination of these chromosomal deletions/gains revealed no specific regions associated with genes previously linked to tumourigenesis. WES revealed on average 10,156 nonsynonymous replacement sites and 15,809 silent sites per tumour type (Supplementary Fig. 3b and Supplementary Data 2). Of the *Tet2*^−/−^ tumours of myeloid, T- or B-cell origin, we found 190 genes with recurrent single-nucleotide variants (SNVs) originating from different tumours. The gene list included *Apc*, *Nf1*, *Flt3*, *Cbl*, *Notch1* and *Mll2* (Fig. 3a and Supplementary Data 3), genes recurrently altered in human haematological malignancies^20^,^21^,^22^,^23^,^24^. The heterodimerization and proline-glutamic acid-serine-threonine-rich domains of NOTCH1 are mutational hotspots in human T-ALL^24^. *Notch1* mutations identified by exome sequencing and Sanger sequencing in *Tet2*^−/−^ T-cell tumours were found within these hotspots (Fig. 3b), suggesting that additional *Notch1* mutations are acquired in *Tet2*^−/−^ mice and contribute to the initiation/progression of the T-cell malignancy.

To confirm that mutations associated with the tumours in aged *Tet2*^−/−^ mice were somatic, we performed additional WES using Lin^−^c-Kit^+^ (LK) cells isolated from premalignant (6-week-old) WT, *Tet2*^*+/−*^ and *Tet2*^−/−^ mice. WES analyses identified few mutations in premalignant LK cells from WT, *Tet2*^*+/−*^ and *Tet2*^−/−^ mice, because genetic changes would only be detectable in a dominant LK cell clone (Supplementary Data 4), so variants identified in *Tet2*^−/−^ tumours are somatic mutations accumulating in *Tet2*^−/−^ cells over time, rather than germline. To overcome this limitation, we performed targeted sequencing on selected loci (from the *Tet2*^−/−^ tumour SNVs) at the single-cell level using WT and premalignant *Tet2*^−/−^ LK cells. Interestingly, we observed a significantly higher frequency of mutations on seven of the 13 selected loci in *Tet2*^−/−^ LK cells compared to WT LK cells (Fig. 3c and Supplementary Table 3), suggesting that *Tet2*^−/−^ LK cells are hypermutagenic.

### Higher mutational burden at sites with gained-5hmC peaks

**Figure 4:**
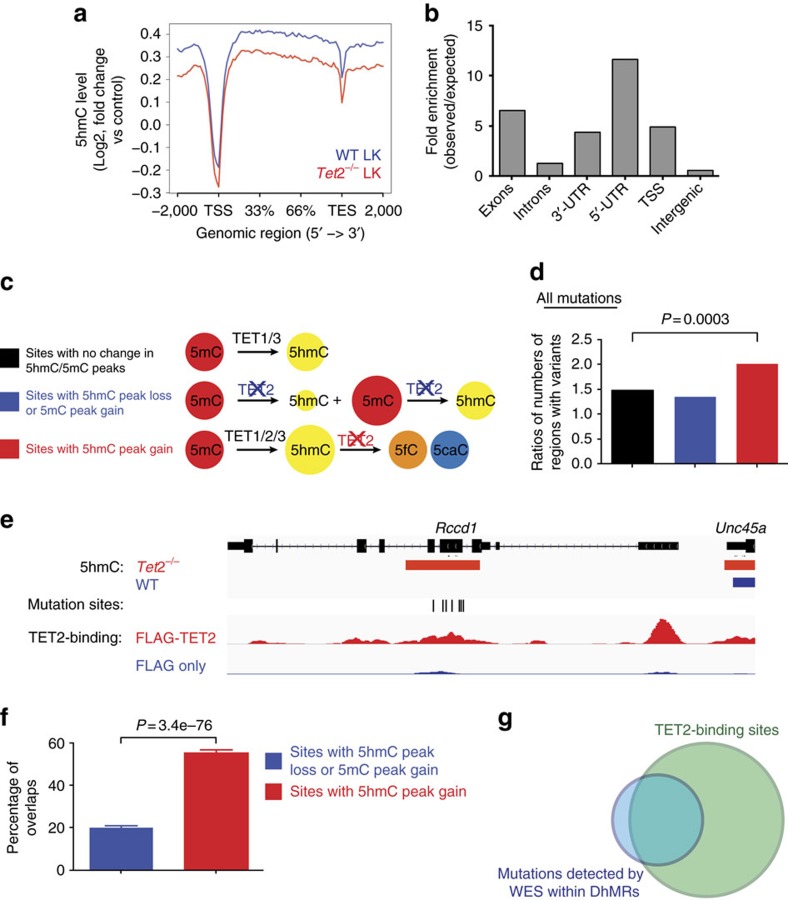
Greater mutational frequencies at loci with 5hmC peak gains in *Tet2*^−/−^ tumours. (**a**) Distribution of average 5hmC enrichment at all genes in WT and *Tet2*^−/−^ LK cells. (**b**) Association of 5hmC-enriched regions with genomic features in LK cells. The percentage of 5hmC-enriched regions overlapping a defined genomic feature was compared to the percentage expected to fall in that particular region by chance based on the genomic coverage of that region. Values are expressed as fold change relative to expected. (**c**) Three categories of 5hmC loci: (1) sites with no change in 5hmC/5mC peaks, (2) sites with 5hmC peak loss or 5mC peak gain wherein TET2 was responsible for the first oxidation step and (3) sites with 5hmC peak gains wherein TET2 was responsible for the second and perhaps third oxidation steps. (**d**,**e**) Significantly greater frequencies of overall SNVs/indels in *Tet2*^−/−^ tumours occur within loci with 5hmC peak gains, but not in loci with no change in 5hmC/5mC peaks and loci with 5hmC peak losses or 5mC peak gains (**d**, *P*=0.0003, *χ*^2^ test). Example genomic view showing overlapping features among the gained-5hmC sites on *Tet2* loss, TET2-binding profile and *Rccd1* mutations (**e**). (**f**) TET2 is enriched more at genomic loci with 5hmC peak gains on *Tet2* loss (*P*=3.4e−76, *χ*^2^ test). Error bars, s.d. (**g**) Venn diagram overlap between TET2-binding sites and SNVs/indels mutations associated with the loss of *Tet2* within DhMRs, as detected by WES.

Given the role of TET proteins in the 5mC oxidation process, we next examined the effect of *Tet2* loss on genome-wide 5hmC and 5mC modification. We applied a selective chemical labelling and affinity enrichment procedure^25^ to map genome-wide 5hmC distributions in premalignant WT and *Tet2*^−/−^ LK cells (Supplementary Data 5). Consistent with previous observations in mouse embryonic stem cells^26^, significant reductions of 5hmC were restricted to bodies of genes intermediately or lowly expressed in LK cells. 5hmC was not affected in highly expressed genes in *Tet2*^−/−^ LK cells (Fig. 4a and Supplementary Fig. 4a). Most TET2-dependent 5hmC modifications in LK cells were located within genes (Fig. 4b). We then explored the overlap between sites with SNVs/indels in *Tet2*^−/−^ tumours and three different genomic loci: (1) sites with no change in 5hmC/5mC peaks, presumably representing the sites with 5mC oxidation catalysed by TET1/3, but not TET2; (2) sites with 5hmC peak loss or 5mC peak gain, representing the 5mC sites that are converted to 5hmC by TET2; and (3) sites with 5hmC peak gain, likely representing the 5hmC sites that are further oxidized to 5fC/5caC by TET2 (Fig. 4c). Using the *χ*^2^ test, consistent with TET2's main role being further oxidation of 5hmC, we saw a significantly greater frequency of SNVs/indels in *Tet2*^−/−^ tumours at loci with 5hmC peak gains compared to loci with no change in 5hmC/5mC peaks and loci with 5hmC peak loss or 5mC peak gain (Fig. 4d,e and Supplementary Fig. 4b,c). Similar increases were also observed with C-to-T (or G-to-A) mutations (Supplementary Fig. 4b). This mutational occurrence at loci with 5hmC peak gain was further confirmed by traditional bisulfite sequencing and TET-assisted bisulfite sequencing at CpG sites with different distances (<30 and >100 bp) to selective mutations (detected by WES) using WT and premalignant *Tet2*^−/−^ LK cells, which showed a significantly higher percentage of CpG sites with a 5hmC gain in sites within 30 bp from the mutation sites (7 out of 10) as compared to sites >100 bp away (1 out of 10, *P*<0.05, by Fisher's exact test) from the mutation sites (Supplementary Fig. 4d,e). Together, loci with 5hmC peak gain on *Tet2* loss are associated with a higher mutational frequency.

We next used chromatin immunoprecipitation sequencing to map genome-wide binding sites of TET2 in MEL (a mouse erythroleukaemia cell line) cells overexpressing FLAG-tagged TET2 (Supplementary Fig. 5a,b and Supplementary Data 6). Genomic analysis revealed that TET2-binding sites are enriched at regions that include exons, 5′-untranslated region and CpG islands (Supplementary Fig. 5c). We analysed the TET2-binding sites with differentially methylated/hydroxymethylated regions in *Tet2*^−/−^ LK cells and observed significant overlap among genes involved in myeloid and B-cell differentiation (Supplementary Fig. 5d,e). In particular, TET2 is enriched more at loci with 5hmC peak gain on *Tet2* loss (Fig. 4f). Furthermore, the majority of mutations we detected within loci that require TET2 for dynamic DNA demethylation overlap with TET2-binding sites (Fig. 4g; 145 out of 212 mutation sites). Collectively, these results suggest that TET2 binds to loci marked by a gain of 5hmC on *TET2* loss, and that at these sites, TET2 may protect genome stability.

### *TET2* loss is associated with increased mutational frequency

**Figure 5:**
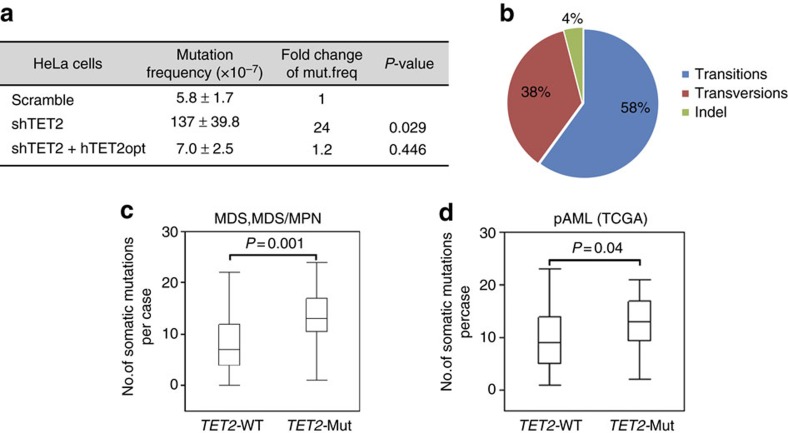
*TET2* loss/mutations are associated with increased mutational frequency and specific mutational spectra. (**a**) *HPRT* mutations in scrambled control-, shTET2- and shTET2+TET2opt HeLa cells. (**b**) Proportions of *Tet2*^−/−^ tumour transitions, transversions and indels. (**c**) Comparison of numbers of nonsynonymous somatic mutations by WES MDS and MDS/MPN cases with (*n*=41) and without (*n*=154) *TET2* mutations (*P*=0.001, Wilcoxon's rank-sum test). (**d**) Comparison of numbers of nonsynonymous somatic mutations in AML cases with and without *TET2* mutations (*P*=0.04, Wilcoxon's rank-sum test) available only for primary AMLs through TCGA. Box plots: upper whisker, maximum value less than 75%+1.5 × IQR; upper box end, 75%; bar, 50%; lower box end, 25%; lower whisker, least value more than 25%−1. × IQR. IQR, interquartile range.

We next examined whether *TET2* loss affects the spontaneous forward mutational frequency in the *hypoxanthine-guanine phosphoribosyltransferase 1* (*HPRT1*) gene in control and *TET2*kd HeLa cells. The *HPRT1* mutational frequency in *TET2*kd HeLa cells increased about 24-fold compared to control HeLa cells (Fig. 5a and Supplementary Fig. 6a,b). Similar results were obtained using control and *Tet2*kd NIH3T3 cells (Supplementary Fig. 6c,d). When we stably re-expressed TET2 using a lentiviral construct containing a mammalian codon-optimized cDNA sequence of *TET2* into *TET2*kd HeLa cells (Supplementary Fig. 6e), *HPRT1* mutational frequencies were completely rescued (Fig. 5a). These results indicate that *TET2* loss causes increases in mutation rates. We then analysed the spectrum of mutations identified in the *HPRT1* gene in 6-thioguanine (6-TG)-resistant clones of *TET2*kd HeLa cells. Interestingly, SNVs and single-nucleotide insertions/deletions (indels) dominated the mutations in the *HPRT1* gene. Approximately 61% and 36% of the mutations in *TET2*kd clones were transitions and transversions, respectively, and ∼3% were indels (Supplementary Fig. 6f). We further analysed the mutational spectrum of *Tet2*^−/−^ tumours. We focussed on mutations located at loci with 5hmC peak gains. Base transition mutations clearly dominated the mutational spectrum in *Tet2*^−/−^ tumours, with G:C to A:T transitions accounting for 35% of total mutations. The remaining mutations consisted primarily of A:T to G:C transitions (23%), various transversions (38%), with G:C to T:A being the most frequent, and +1/−1 indels (4%), with −1 deletions being more common than +1 (Fig. 5b). Of all mutations isolated, 73% occurred at CpG sites or clustered around CpG sites (±30 bp); since the average distance between CpG sites across the genome is 100 bp^27^,^28^, <60% was expected in the gene bodies. The close association of mutation sites with CpG sites suggests a preference of mutational occurrence at or around CpG sites in *Tet2*^−/−^ tumours. The hypermutagenicity and mutational spectra of *TET2*kd and *Tet2*^−/−^ cells and the function of TET2/TDG in 5mC oxidation and demethylation suggest that TET2 is likely involved in safeguarding genomic mutagenicity.

We further analysed a large cohort of MDS and MDS/MPN patients (Supplementary Table 4) for the presence of somatic mutations using exome sequencing of paired samples and targeted deep sequencing of 60 genes (including *TET2*) commonly mutated in these conditions (Supplementary Data 7). Exome analysis revealed that patients with *TET2* mutations harbour a significantly higher number of mutational events compared to those with WT *TET2* (Fig. 5c). Similar results were obtained in AML patient data when the The Cancer Genome Atlas (TCGA) leukaemia cohort was analysed (Fig. 5d). Of the 556 mutations identified in MDS and MDS/MPN patients with *TET2* mutations, 52% and 12% were transitions and indels, respectively (Supplementary Fig. 6g), slightly higher than in patients with WT *TET2* (Supplementary Fig. 6h). Confirmatory deep sequencing of a combined cohort showed that somatic *TET2* mutations were associated with other subclonal events, chiefly in *APC*, *NF1*, *ASXL1*, *CBL* and *ZRSR2*. Consistently, many of these genes (for example, *Apc*, *Nf1* and *Cbl*) are also affected by subclonal mutations in *Tet2*^−/−^ mice (Fig. 3a). Increases in numbers of subclonal events in patients with *TET2* mutations are consistent with *Tet2* loss leading to genome-wide hypermutability in HSCs/HPCs.

## Discussion

In this study we show that, in addition to myeloid malignancies, a fraction of *Tet2*^−/−^ mice develop T- and B-cell malignancies. These findings are consistent with clinical observations that loss-of-function mutations in *TET2* arise not only in human myeloid malignancies but also in subtypes of B- and T-cell malignancies^6^,^7^,^8^,^9^,^10^,^11^,^16^. Therefore, our *Tet2*^−/−^ mice can serve as a model for investigations of mechanisms by which *Tet2* loss leads to diverse haematological malignancies. Leukaemogenesis is a multistep process of acquiring gene mutations. Loss-of-function *TET2* mutations are initiating events in the pathogenesis of haematological malignancies. The occurrence of secondary oncogenic gene mutations, presumably in an early haematopoietic progenitor, is likely capable of modifying the disease phenotype and/or promoting the transformation/progression of a specific haematological malignancy in *Tet2*^−/−^ mice. WES identified numerous SNVs/indels in *Tet2*^−/−^ tumours, indicating that *TET2* loss constitutes a state of genetic mutagenicity. Consistently, the number of mutational events appears to be higher in MDS, MDS/MPN and primary AML patients harbouring *TET2* mutations compared to those with WT *TET2*. However, this observation from patient exome analysis does not necessarily imply a direct role of *TET2* mutations in promoting additional mutations, since other factors associated with patients with *TET2* mutations might also contribute to this observation such as older ages, increased stem cell proliferation and/or longer average disease latency due to a preceding phase of clonal haematopoiesis. The increased susceptibility to mutation acquisitions is likely further accelerated in *TET2-*deficient HSCs/HPCs since *Tet2* loss induced intrinsic changes in these cells, with increased self-renewal and proliferation. Our study identified mutations on the genes implicated in the pathogenesis of the diverse disease phenotypes in *Tet2*^−/−^ mice. Mutations in *Notch1*, *Flt3*, *Nf1*, *Ebf1*, *Apc*, *Cbl* and other genes in the *Tet2*^−/−^ haematological malignancies are consistent with human haematological malignancy gene alterations^20^,^21^,^22^,^23^,^24^. Recent studies have shown that *TET2* mutations also occur in healthy elderly individuals with clonal haematopoiesis^13^. Therefore, as an ancestral event in haematological malignancies and elderly individuals, *TET2* mutations represent a suitable target for intervention at the early stages of clonal expansion of HSCs/HPCs.

Although we have shown that the catalytic activity of TET2 is essential for its myeloid tumour-suppressive function in HSCs/HPCs^19^, our recent RNA-seq and 5mC/5hmC analyses on premalignant *Tet2*^−/−^ and WT LK cells showed that distinct cytosine modifications (particularly 5hmC) often mark specific genes in *Tet2*^−/−^ LK cells without altering their expression^17^. The oxidation and demethylation of 5mC in the genome are regulated in a sophisticated manner. 5fC and 5caC are substrates for TDG^1^,^29^. TETs and TDG initiate active DNA demethylation by oxidation of 5mC and excision of 5fC/5caC in DNA, followed by incision and repair of the resulting abasic site (AP site). Although the BER process is highly accurate, errors may occur due to replication across 5mC derivatives and due to polymerase errors during gap filling. Indeed, a recent *in vitro* biochemical study showed that TET1 and TDG mediate sequential demethylation of symmetrically methylated CpGs to avoid DNA double-strand breaks, which could kill or transform the cells^30^. 5mC is found to be a mutagen in tumour cells^31^. In addition, a recent report finds that ‘G'-5caC base pairs mimic mismatches during DNA replication^32^. Thus, genomic 5mC derivatives should normally be processed error free by BER, with mismatch repair likely serving as a ‘backup' for certain 5mC oxidation derivatives during DNA replication.

Mutations are generally considered to occur randomly throughout the genome. Although we did not observe an overt base preference in *Tet2*^−/−^ tumours mutations, the mutations did tend to be proximal to CpG sites. Our study also demonstrated that a significantly higher frequency of mutations occurred at loci with the gain of 5hmC in *Tet2*^−/−^ tumours, where TET2 normally binds. The mechanisms that govern the association of *Tet2* loss with increased local mutation rates at/around gained-5hmC sites remain to be determined. A recent study showed that *Ung*^−/−^ mice exhibit higher spontaneous mutation rates and increases in spontaneous B-cell lymphomas^33^. Furthermore, *Mbd4*^−/−^ mice were recently characterized as having increased rates of C-to-T transitions at CpG sites^34^. In this backdrop, our studies suggest that *TET2* loss and *TET2* loss-mediated 5mC oxidation dysregulation are associated with increased mutagenicity at specific genomic loci. Thus, TET2 protects HSC/HPC genomes against mutagenicity. There are now genomic profiling methods for 5fC and 5caC^35^,^36^,^37^. Given that 5fC and 5caC are directly excised by TDG/BER during active demethylation, it will be interesting to see whether sites with alterations in 5fC and 5caC marks in *Tet2*^−/−^ LK cells are preferentially associated with higher mutation rates. Parallel, genome-wide, base-resolution maps of 5mC, 5hmC, 5fC and 5caC in *Tet2*^−/−^ and WT LK cells are warranted for dissecting TET2-mediated genome-wide DNA demethylation dynamics in HSCs/HPCs and for uncovering correlations between specific 5mC, 5hmC, 5fC or 5caC alterations and the genomic hypermutagenicity caused by *Tet2* loss. Indeed, consistent with our results, a recent report provides evidence that, more than TET1 or 3, a forte of TET2 is the further oxidation of 5hmC^38^.

In summary, we show that *TET2* loss leads to hypermutagenicity in HSCs/HPCs, preferentially at loci with the gain of 5hmC on the loss of TET2, where TET2 normally binds. TET2-dependent 5hmC marks are highly enriched at gene bodies, such as exons of LK cells. *TET2*-deficient HSCs/HPCs that become hypermutagenic are likely not malignant *per se*, but higher mutation rates in these cells may result in additional driver mutation(s) in TET2 target genes over time. Such states may be amenable to TET2 activity-boosting chemoprevention approaches. Our results unveil a novel role for TET2 in safeguarding genome mutagenicity and provide additional insights into the mechanisms by which loss-of-function TET2 mutations cause diverse human haematological malignancies. Further mechanistic studies are needed to determine how *TET2* loss leads to increased DNA mutagenicity in HSCs/HPCs and thus the increased risks of haematopoietic malignancies.

## Methods

### Analyses of mice

*Tet2*-knockout (*Tet2*^−/−^) mice were generated as described^14^. Animal care was conducted in accordance with institutional guidelines and approved by the Institutional Animal Care and Use Committee (IACUC), University of Miami Miller School of Medicine. PB was collected by retro-orbital bleeding of mice and was smeared for May–Grünwald–Giemsa staining, and/or subjected to an automated blood count (Hemavet System 950FS). For histopathology analyses, femurs were fixed in formaldehyde, decalcified and paraffin embedded. Spleens, livers, lymph nodes, thymus and intestine were treated similarly, except for the decalcification step. Sections (4.5 μm) were stained with haematoxylin and eosin (H&E). For flow cytometric analyses, single-cell suspensions from BM, spleen, liver, lymph node, thymus and PB were stained with panels of fluorochrome-conjugated antibodies. Dead cells were excluded by 4,6-diamidino-2-phenylindole staining. Analyses were performed using a BD FACSCanto II or LSRII flow cytometer. All data were analysed by FlowJo7.6 software.

### Mouse exome sequencing

Initial WES was carried out to identify candidate mutations in the exome of genes. Genomic DNA was captured with the NimbleGen mouse exome array according to the manufacturer's protocol, and 100-bp paired-end sequencing was performed using an Illumina HiSeq 2000. Raw sequencing reads were mapped to the whole mouse genome (mm10) using PEMapper/PECaller (https://github.com/wingolab-org/pecaller) with the default settings^39^, and variant bases were annotated with SeqAnt (http://seqant.genetics.emory.edu/)^40^. For samples subjected to mutation detection, genomic DNA was amplified in selected exons by PCR (primers shown in Supplementary Data 8) and sequenced by Sanger sequencing. The location and types of mutations were then determined by sequencing results.

### *HPRT* mutation analyses

Mutation analyses can be achieved in proliferating cells *in vitro* by anHPRT1 assay that positively selects for HPRT-deficient mutants based on their resistance to 6-TG, which is lethal to HPRT-WT cells that are proficient in free purine base salvage^41^. The *HPRT* mutation assay was conducted as described previously^42^. Briefly, cells (5 × 10^5^) were seeded in triplicate in 10-cm petri dishes for 12 h and fed with complete medium containing 5 μM freshly prepared 6-TG. Plating efficiency was determined by culturing 5 × 10^2^ cells in the absence of 6-TG. After 10 days of culturing, colonies were visualized by staining with 0.05% crystal violet. The mutation frequency was then the ratio of the number of clones in the presence of 6-TG to the total number of cells plated, normalized by the plating efficiency. Types of mutations were characterized by DNA sequencing coding regions of the *HPRT* gene using primers shown in Supplementary Data 8.

### Patients

The mutational statuses for *TET2* and other coexisting genes were analysed in BM and blood specimens from patients with various myeloid neoplasms, including MDS, MDS/MPN and secondary AML (see Supplementary Table 4 and Supplementary Data 5). Informed consent was obtained according to protocols approved by the institutional review boards and in accordance with the Declaration of Helsinki. Diagnosis was confirmed at each institution according to the World Health Organization classification criteria. Analysis of TCGA primary AML cases was performed using publically available data sets (http://tcga-data.nci.nih.gov/tcga/tcgaHome2.jsp).

### WES for human patient samples

WES and targeted capture sequencing were performed as described previously^43^. For WES, the 50 Mb of protein coding sequences was enriched from total genomic DNA by liquid-phase hybridization using SureSelect (version 4) (Agilent Technology), followed by massively parallel sequencing with HiSequation 2000 (Illumina). Somatic mutations were detected using our in-house pipeline, followed by validation using amplicon deep sequencing^43^,^44^. To minimize false positives and focus on the most prevalent or relevant somatic events, we implemented a rational bioanalytic filtering approach and applied heuristic bioanalytic pipelines. We used two independent pipelines to identify somatic and germline alterations. For confirmation of somatic mutations, we analysed paired germline DNA from CD3^+^ lymphocytes. The selected observations were validated by targeted deep sequencing using MiSeq. Our sequence library for deep sequencing was generated by TruSeqCustom Amplicon (Illumina).

### Targeted multiamplicon deep sequencing of patient samples

We applied multiamplicon-targeted deep sequencing (TrueSeq; Illumina) to frequently affected exons of 60 selected genes^45^. The sequencing libraries were generated according to an Illumina paired-end library protocol and subjected to deep sequencing on MiSeq (Illumina) instrumentation according to standard protocol. High-probability oncogenic mutations were called by eliminating sequencing/mapping errors and known/possible single-nucleotide polymorphisms based on available databases and frequencies of variant reads. Genomic copy number status was calculated by directly enumerating corresponding sequencing reads in each exon.

### Statistical analysis

Differences between experimental groups were determined by the Student's *t*-test, Fisher's exact test, Wilcoxon's rank-sum test and/or analysis of variance, followed by Newman–Keuls multiple comparison tests as appropriate. *P* values <0.05 were considered significant. For SNV count data, *χ*^2^ tests were used as implemented in R (http://cran.r-project.org/)^46^.

### Data availability

Genome-wide data sets generated for this study are deposited at GEO under the accession number GSE74390. All other remaining data are available within the article and Supplementary Files, or available from the authors on request.

## Additional information

**How to cite this article:** Pan, F. *et al. Tet2* loss leads to hypermutagenicity in haematopoietic stem/progenitor cells. *Nat. Commun.* **8,** 15102 doi: 10.1038/ncomms15102 (2017).

**Publisher's note:** Springer Nature remains neutral with regard to jurisdictional claims in published maps and institutional affiliations.

## Supplementary Material

Supplementary Information Supplementary Figures, Supplementary Tables, Supplementary Methods and Supplementary References

Supplementary Data 1 aCGH by tumor type.

Supplementary Data 2 List of the counts of unique variants by Tet2-/- tumor type.

Supplementary Data 3 List of genes with recurrent SNVs and genes with recurrent missense in Tet2-/- tumors.

Supplementary Data 4 WES from premalignant young mice.

Supplementary Data 5 Genome-wide 5hmC distributions in premalignant WT and Tet2-/- LK cells.

Supplementary Data 6 TET2 ChIP-seq data in MEL cells stably expressing FLAG-mTET2opt.

Supplementary Data 7 Mutations in TET2-wt and TET2-mut MDS and MDS/MPN patients.

Supplementary Data 8 Primers used in the study.
