# Supplementary material for: Tet2 loss leads to hypermutagenicity in haematopoietic stem/progenitor cells
Source: Nat Commun. 2017 Apr 25;8:15102. doi: 10.1038/ncomms15102 (PMC5414116; doi:10.1038/ncomms15102)
Supplement: Supplementary Information — Supplementary Figures, Supplementary Tables, Supplementary Methods and Supplementary References [file ncomms15102-s1.pdf]

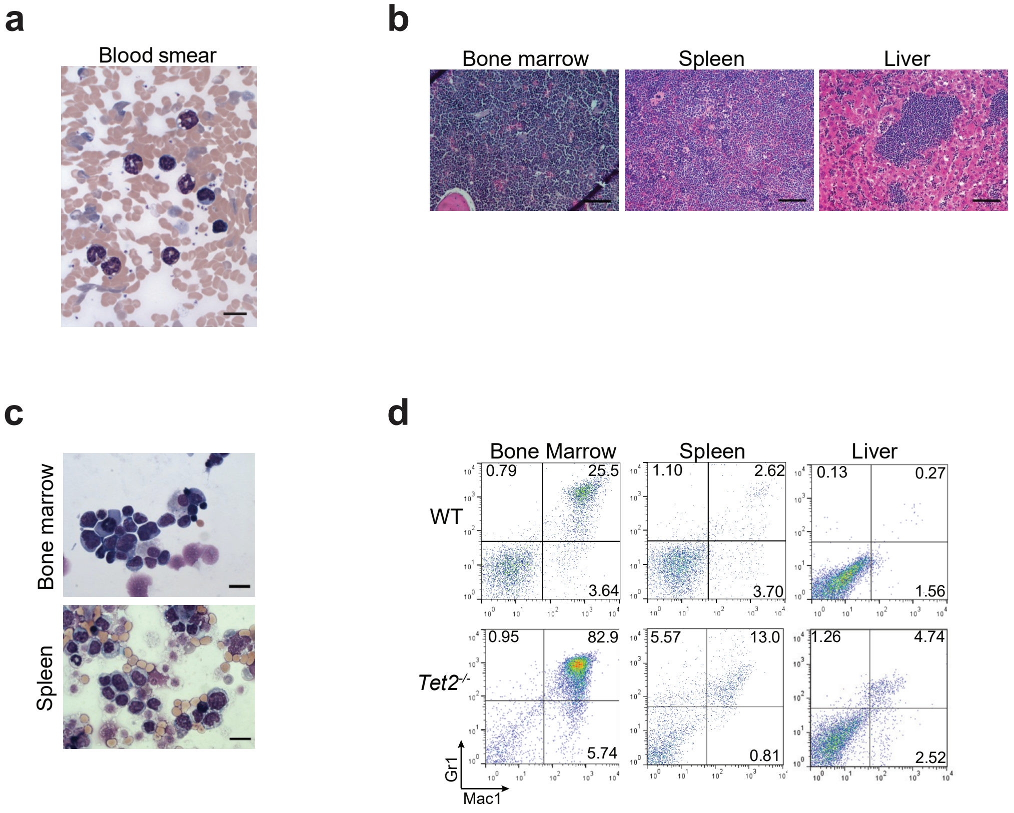


**Supplementary Figure 1. *Tet2*$^{-/-}$ mice develop myeloid malignancies.**
(a) May-Grünwald Giemsa stained peripheral blood smears prepared from a representative *Tet2*$^{-/-}$ mouse with myeloid malignancy. Scale bar, 20 um.
(b) H&E stained histological sections of femur (bone marrow), spleen and liver from a representative *Tet2*$^{-/-}$ mouse with myeloid malignancy. Bone marrow and spleen exhibited increased myeloid precursors and monocytes. Liver showed nodular and sinusoidal myeloid cell infiltration. Scale bar, 12.5 um.
(c) May-Grünwald Giemsa stained bone marrow and spleen cytospin preparations from a representative *Tet2*$^{-/-}$ mouse with myeloid malignancy. Scale bar, 20 um.
(d) Flow cytometric analysis of the myeloid lineage (Gr1/Mac1) in bone marrow, spleen and liver of a representative *Tet2*$^{-/-}$ mouse with myeloid malignancy.

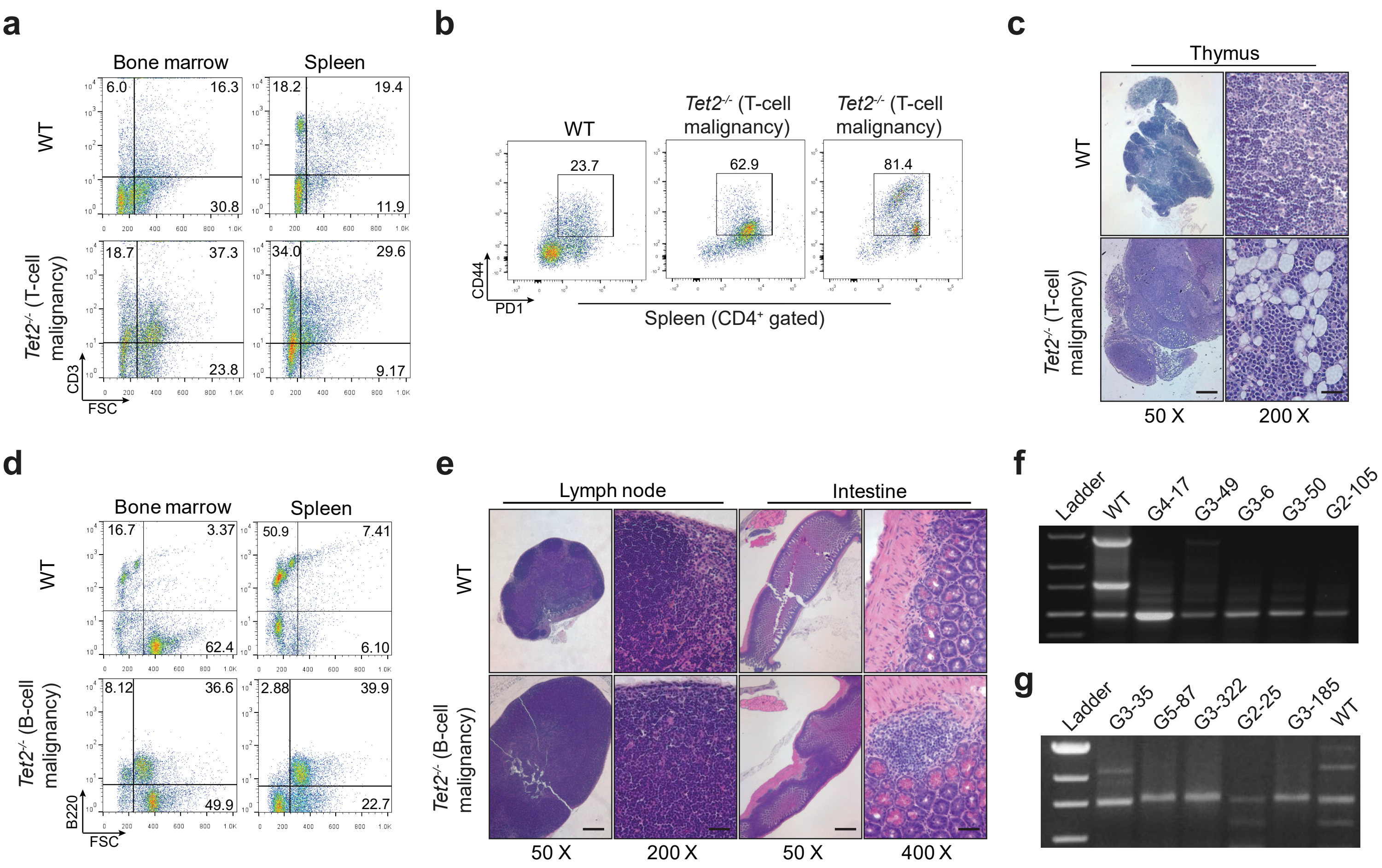


**Supplementary Figure 2.** ***Tet2*$^{-/-}$ mice develop lymphoid malignancies.**
(a) Flow cytometric analysis of the T-cell lineage (CD3/FSC) in bone marrow and spleen of a representative *Tet2*$^{-/-}$ mouse with T-cell malignancy and an age-matched WT mouse.
(b) Flow cytometric analysis of the CD4$^{+}$ T-cells (CD44/PD1) in spleen of representative *Tet2*$^{-/-}$ mice with T-cell malignancy and an age-matched WT mouse.
(c) H&E stained histological sections of thymus from a representative *Tet2*$^{-/-}$ mouse with T-cell malignancy and an age-matched WT mouse. Scale bar: 50 X, 100 um; 200 X, 25 um.
(d) Flow cytometric analysis of the B-cell lineage (B220/FSC) in BM and spleen of a representative *Tet2*$^{-/-}$ mouse with B-cell malignancy and an age-matched WT mouse.
(e) H&E stained histological sections of lymph node and intestine from a representative *Tet2*$^{-/-}$ mouse with B-cell malignancy and an age-matched WT mouse. Scale bar: 50 X, 100 um; 200 X, 25 um; 400 X, 12.5um.
(f) Clonal T-cell expansions are identified by *TCRγ* gene rearrangements in splenic CD3$^{+}$ cells from 5 *Tet2*$^{-/-}$ mice with T-cell malignancy.
(g) Clonal B-cell expansions are identified by IgH D-J rearrangement in splenic B220$^{+}$ cells from 5 *Tet2*$^{-/-}$ mice with B-cell malignancy.

**a**

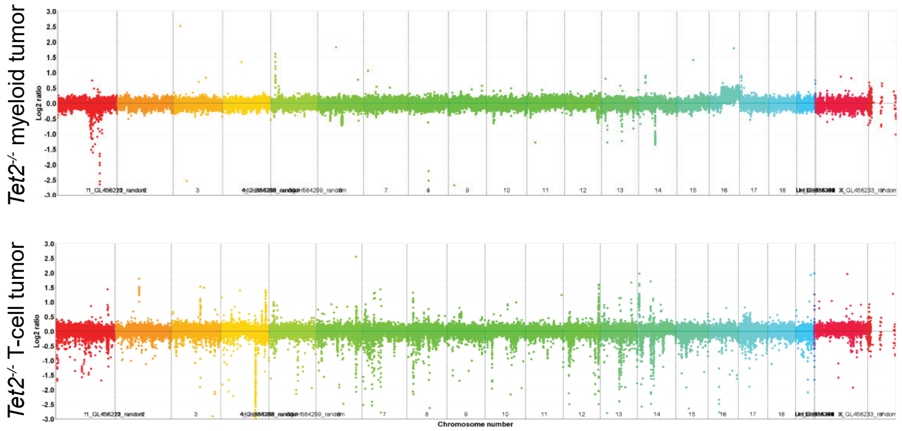

**b**

| Tumor Type | Indel (Exonic) | | SNV (Exonic) | | Silent:Replacement | Transitions:Transversions | Genes with Recurrent Replacement Sites Unique to Tumor Cells |
|---|---|---|---|---|---|---|---|
| | FrameShift | InFrame | Replacement | Silent | | | |
| Myeloid | 151 | 78 | 10306 | 16194 | 1.57 | 2.55 | 515 |
| Myeloid | 192 | 101 | 11095 | 17193 | 1.55 | 2.45 | 613 |
| Myeloid | 158 | 90 | 10463 | 16791 | 1.60 | 2.56 | 452 |
| Myeloid | 164 | 92 | 10881 | 16958 | 1.56 | 2.55 | 645 |
| Myeloid | 151 | 86 | 10686 | 16723 | 1.56 | 2.58 | 717 |
| Myeloid | 143 | 89 | 10659 | 16744 | 1.57 | 2.61 | 41 |
| Myeloid | 170 | 90 | 10667 | 16507 | 1.55 | 2.53 | 480 |
| T-cell (G4-17) | 147 | 83 | 10505 | 16713 | 1.59 | 2.55 | 152 |
| T-cell (G3-1) | 148 | 87 | 10746 | 16670 | 1.55 | 2.47 | 596 |
| T-cell (G3-6) | 163 | 96 | 10945 | 16940 | 1.55 | 2.56 | 753 |
| B-cell (G3-185) | 160 | 93 | 10766 | 16511 | 1.53 | 2.53 | 380 |
| B-cell (G3-38) | 83 | 60 | 6817 | 10350 | 1.52 | 2.63 | 210 |
| B-cell (G5-87) | 81 | 62 | 7492 | 11228 | 1.50 | 2.59 | 266 |

**Supplementary Figure 3. Genetic alterations in *Tet2*$^{-/-}$ tumors.**
(a) Array-comparative genomic hybridization (aCGH) copy number profiles of representative myeloid (upper) and T-cell (lower) tumors from *Tet2*$^{-/-}$ mice showing numerical imbalances. The X-axis represents the genomic position; the Y-axis represents the normalized log2 hybridization ratios.
(b) Summary of SNVs and Indels in *Tet2*$^{-/-}$ tumors detected by WES.

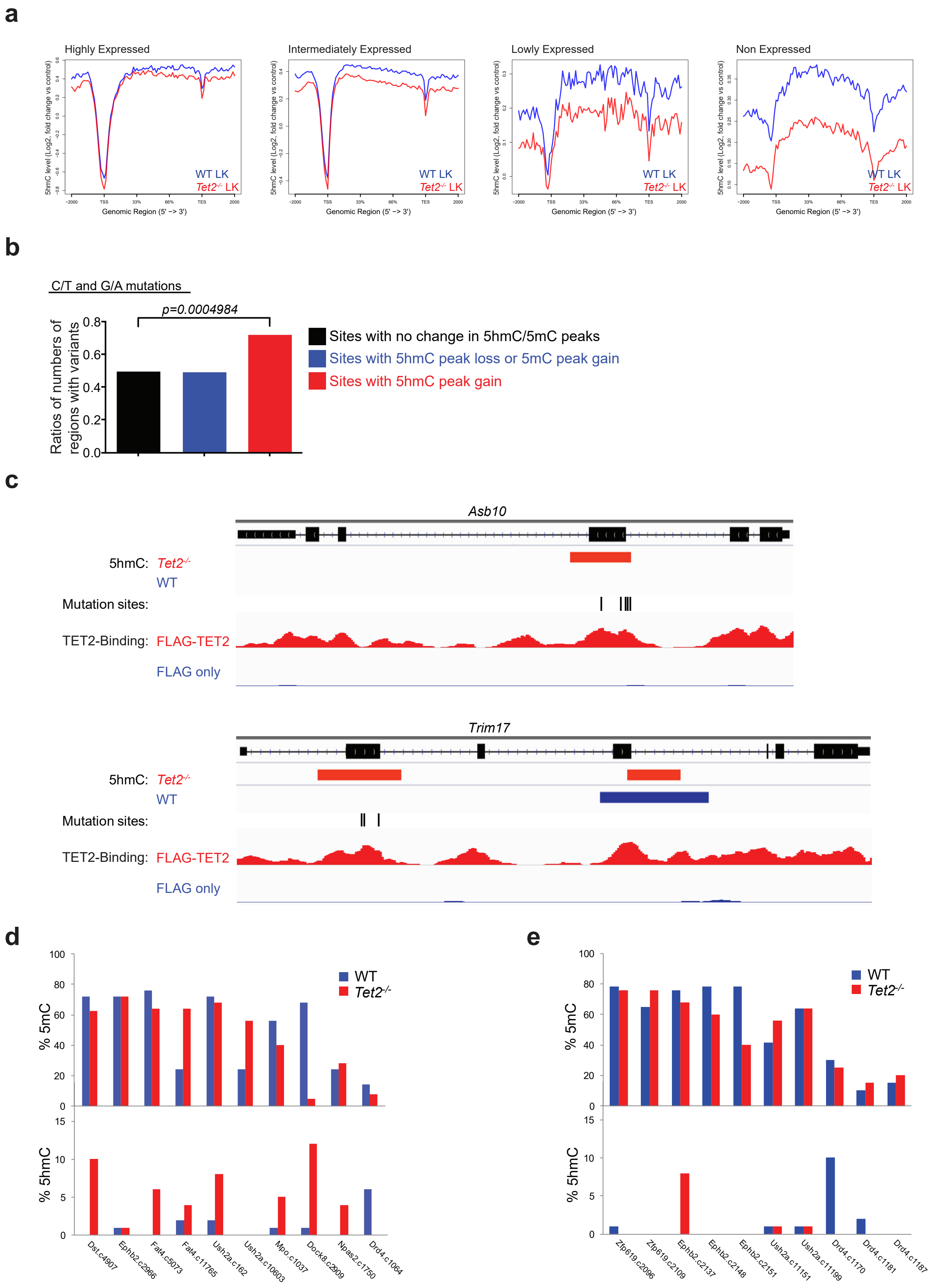
a
Highly Expressed
Intermediately Expressed
Lowly Expressed
Non Expressed
5hmC level (Log2, fold change vs control)
WT LK
Tet2-/- LK
Genomic Region (5' -> 3')
-2000
TSS
33%
66%
TES
2000
b
C/T and G/A mutations
p=0.0004984
Ratios of numbers of regions with variants
0.0
0.2
0.4
0.6
0.8
Sites with no change in 5hmC/5mC peaks
Sites with 5hmC peak loss or 5mC peak gain
Sites with 5hmC peak gain
c
Asb10
5hmC: Tet2-/-
WT
Mutation sites:
TET2-Binding: FLAG-TET2
FLAG only
Trim17
5hmC: Tet2-/-
WT
Mutation sites:
TET2-Binding: FLAG-TET2
FLAG only
d
% 5mC
% 5hmC
WT
Tet2-/-
Dst.c4907
Ephb2.c2966
Fat4.c5073
Fat4.c11765
Ush2a.c162
Ush2a.c10603
Mpo.c1037
Dock8.c2909
Npas2.c1750
Drd4.c1064
e
% 5mC
% 5hmC
WT
Tet2-/-
Zfp619.c2096
Zfp619.c2109
Ephb2.c2137
Ephb2.c2148
Ephb2.c2151
Ush2a.c11151
Ush2a.c11199
Drd4.c1170
Drd4.c1181
Drd4.c1187

**Supplementary Figure 4. Overlapping features of mutation sites in *Tet2*$^{-/-}$ tumor cells and sites with 5hmC peak gain.**

(a) Gene expression values (RPKM) obtained from RNA-seq were used to divide all the genes into four groups: Highly expressed (top 25%), Intermediately expressed (25-50%), Lowly expressed (50-75%) and Non-expressed (below 75%). Distributions of the averaged 5hmC enrichment at all genes per group in WT and *Tet2*$^{-/-}$ LK cells are shown.
(b) Significantly greater frequencies of C->T and G->A SNVs are found within loci with 5hmC peak gain relative to regions with 5hmC peak loss or 5mC peak gain or no change in 5hmC/5mC peaks ($p$=0.0004984, chi-square test).
(c) Representative genes (*Asb10* and *Trim17*) showing overlap among mutation sites and sites with 5hmC peak gain and TET2-binding profile.
(d) Analysis of the levels of 5mC and 5hmC by Bisulfite-seq and TAB-seq in CpG sites within 30bp of selected mutations (from WES of *Tet2*$^{-/-}$ tumors) in WT and premalignant *Tet2*$^{-/-}$ LK cells.
(e) Analysis of the levels of 5mC and 5hmC by Bisulfite-seq and TAB-seq in CpG sites >100bp away from selected mutations (from WES of *Tet2*$^{-/-}$ tumors) in WT and premalignant *Tet2*$^{-/-}$ LK cells. Significantly greater percentage of CpG sites with a 5hmC gain upon *Tet2* loss were observed in the CpG sites within 30bp of mutations as compared to the CpG sites >100bp away from mutations (7 out of 10 v.s. 1 out of 10; $p$=0.0198, Fisher's exact test).

a

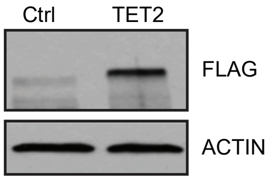

Ctrl
TET2
FLAG
ACTIN


b

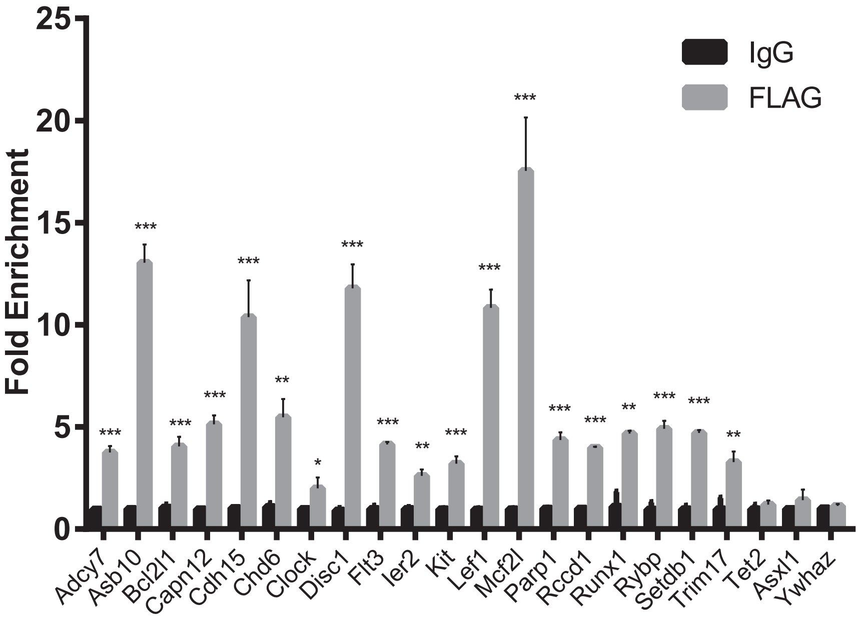

Fold Enrichment
IgG
FLAG
Adcy7
Asb10
Bcl2l1
Capn12
Cdh15
Chd6
Clock
Disc1
Flt3
Ier2
Kit
Lef1
Mcf2l
Parp1
Rccd1
Runx1
Rybp
Setdb1
Trim17
Tet2
Asxl1
Ywhaz


c

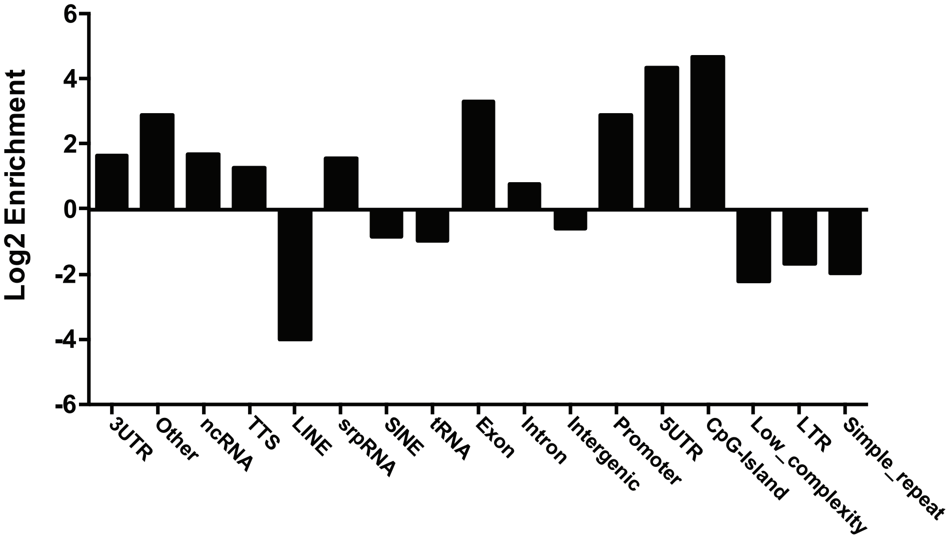

Log2 Enrichment
3UTR
Other
ncRNA
TTS
LINE
srpRNA
SINE
tRNA
Exon
Intron
Intergenic
Promoter
5UTR
CpG-Island
Low_complexity
LTR
Simple_repeat

d

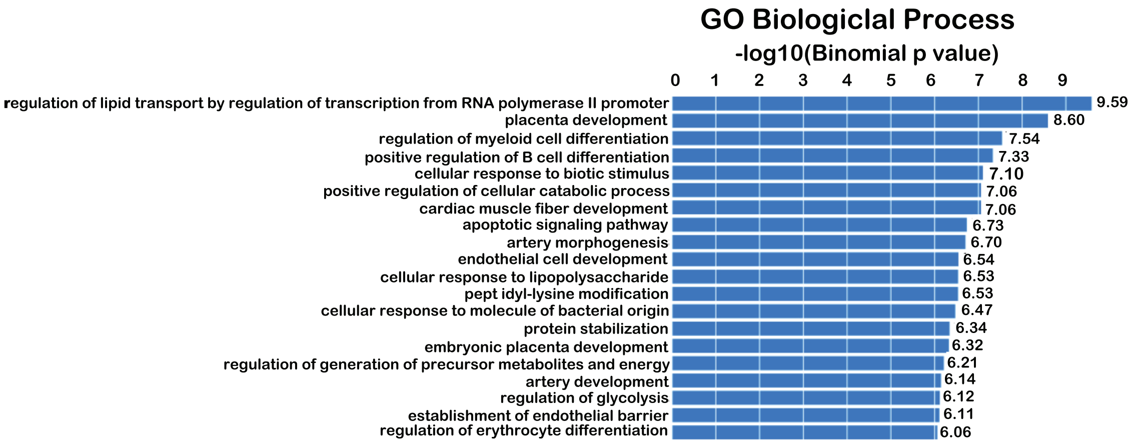


e

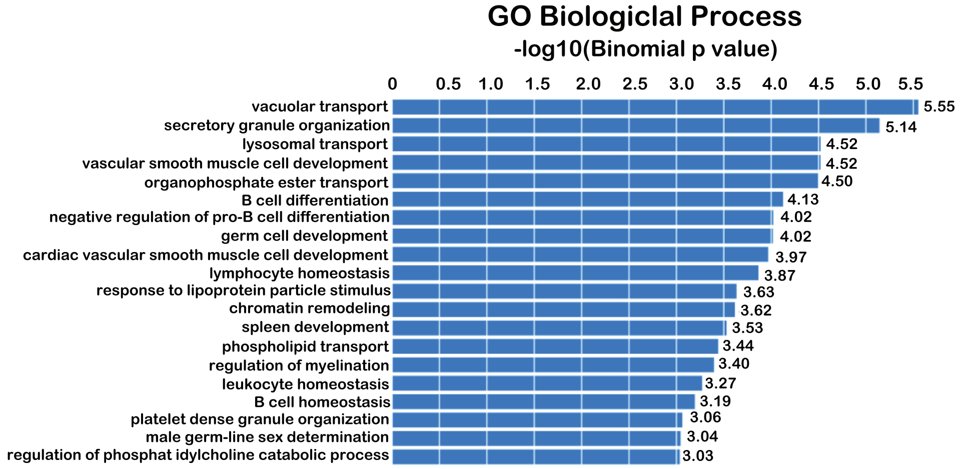


**Supplementary Figure 5. TET2-binding sites are enriched at gene bodies.**
(a) Western blot analysis shows the expression of FLAG-TET2 in MEL cells stably transduced with lentivirus encoding mouse FLAG-TET2.
(b) ChIP-qPCR showing FLAG-TET2 (FLAG) enrichment relative to IgG in 19 regions derived from the identified FLAG-TET2 binding sites and 3 control regions (*Tet2*, *Asxl1* and *Ywhaz*) >2k bp away from the FLAG-TET2 binding sites in MEL cells expressing FLAG-TET2 (three independent experiments; means±S.D.). *$p$<0.05, **$p$<0.01, ***$p$<0.001 (Student's t-test).
(c) Genomic feature annotation of TET2-binding sites reveals strong enrichment of TET2 in exons, 5' UTRs and CpG islands relative to genomic background.
(d) Gene ontology analyses of the overlapping genes between TET2-binding sites and DhMR specifically associated with WT LK cells. GO analysis was performed using GREAT with WT-specific DhMRs. Most significant GO biological processes are indicated in the bar graph.
(e) Gene ontology analyses of the overlapping genes between TET2-binding sites and DhMR specifically associated with $Tet2^{-/-}$ LK cells. GO analysis was performed using GREAT with KO-specific DhMRs. Most significant GO biological processes are indicated in the bar graph.

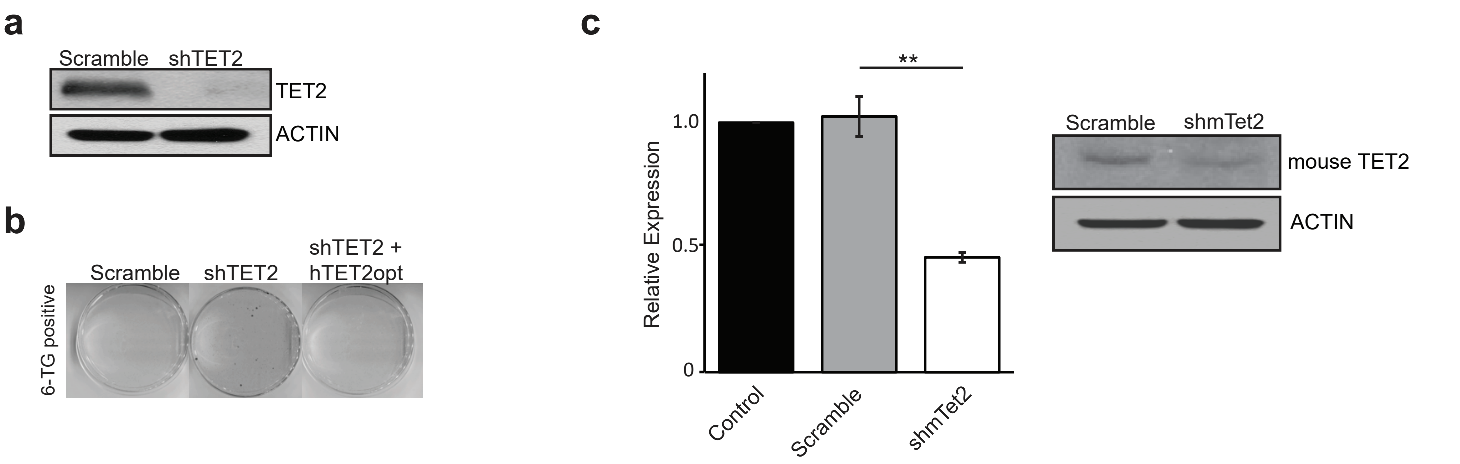


d

| NIH3T3 Cells | Mutation Frequency ($x10^{-7}$) | Fold change of Mut.Freq. | *p* value |
|---|---|---|---|
| Scramble shRNAs | 9.3±1.6 | 1 | 0.027 |
| mTet2 shRNA | 142.9±22.5 | 15 | |

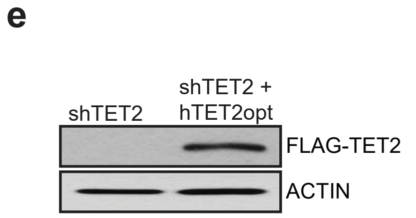


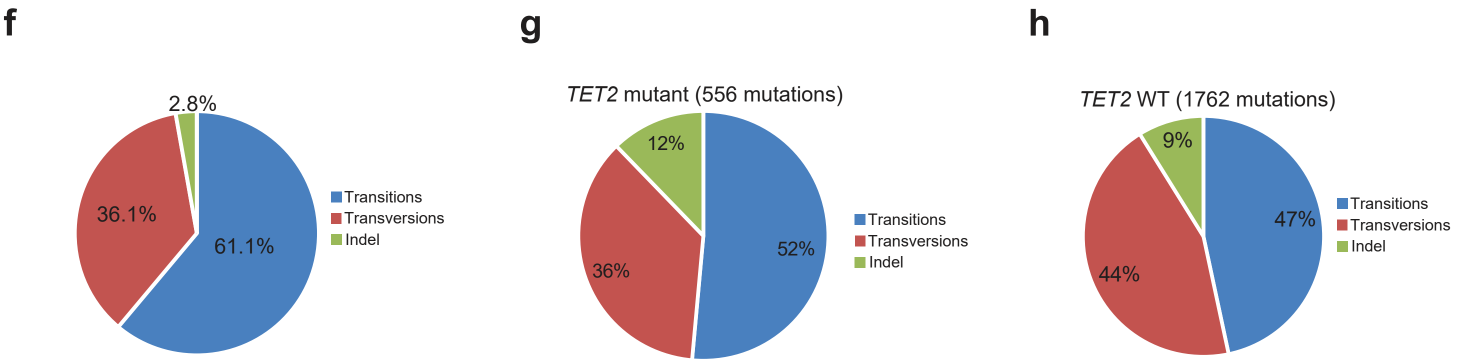


**Supplementary Figure 6. Effects of *Tet2* loss on various DNA repair pathways.**
(a) TET2 expression levels in scrambled control- and shTET2-HeLa cells.
(b) HPRT assays of HeLa cells infected with scramble shRNA, TET2 shRNA and *TET2*kd HeLa cells stably expressing FLAG-TET2opt, stained after growth as indicated.
(c) Western blot (right) and qPCR (left) analysis showing the expression of TET2 mRNA and protein in NIH3T3 cells stably transfected with a scrambled shRNA or a Tet2-specific shRNA. Means±S.D. are shown. ***p*<0.01 (Student's t-test).
(d) *Hprt* mutability in control- and shTet2-NIH3T3 cells. Data are presented as mean ± SD. Fold increase in mutation frequency was calculated using the mutation frequency of control NIH3T3 cells as a reference, and a t-test was used to estimate the *P* value.
(e) Western blot analysis showing the expression of FLAG-TET2opt in *TET2*kd HeLa cells stably expressing FLAG-TET2opt.
(f) Spectra of mutations identified in the *HPRT1* gene in 6-TG resistant clones from shTET2-HeLa cells.
(g) The mutational spectra (in transitions, transversions and indel categories) of non-synonymous somatic mutations in MDS and MDS/MPN cases with *TET2* mutations (n=41).
(h) The mutational spectra (in transitions, transversions and indel categories) of non-synonymous somatic mutations in MDS and MDS/MPN cases with WT *TET2* (n=154).

**Supplementary Figure 7. Full scan images of gel electrophoresis.**

SuppFigure 5a

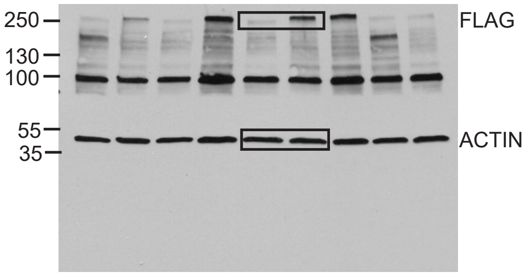


SuppFigure 6b

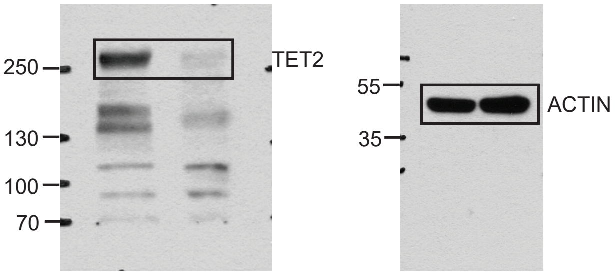


SuppFigure 6c

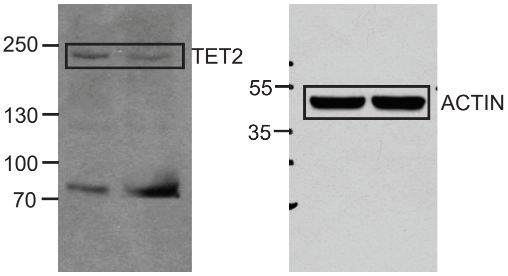


SuppFigure 6e

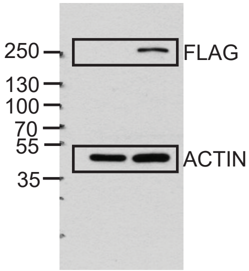

**Supplementary Table 1. Phenotypic characteristics of the T-cell malignancy developed in 7 *Tet2*$^{-/-}$ mice.**

| ID | Age, days | Necropsy findings | Thymus weight (g) | Hematopoietic Disease Phenotype | Blood Counts | | | TCR Rearrangement | Diagnosis |
|---|---|---|---|---|---|---|---|---|---|
| | | | | | WBC, K/μL | LY, K/μL | RBC, M/μL | | |
| G3-1 | 615 | Hepatosplenomegaly, Lymphadenopathy | 0.201 | $CD3^+CD4^+CD8^-$ $CD44^+PD1^+$ | 11.7 | 10.1 | 7.48 | N/A | PTCL |
| G4-17 | 105 | Hepatosplenomegaly, Lymphadenopathy | 0.347 | $CD3^+CD4^+CD8^-$ $CD44^+PD1^+$ | | N/D | | Clonal | PTCL |
| G2-105 | 440 | Hepatosplenomegaly, Lymphadenopathy | 0.300 | $CD3^+CD4^+CD8^-$ $CD44^+PD1^+$ | 15.5 | 13.3 | 7.59 | Clonal | PTCL |
| G3-49 | 381 | Hepatosplenomegaly | 0.056 (Normal) | $CD3^+CD4^+CD8^-$ $CD44^+PD1^+$ | 18.5 | 11.7 | 5.65 | Clonal | PTCL |
| G3-24 | 305 | Hepatosplenomegaly | 0.238 | $CD3^+CD4^+CD8^-$ $CD44^+PD1^+$ | 9.7 | 5.5 | 8.61 | N/A | PTCL |
| G3-6 | 200 | Hepatosplenomegaly, Lymphadenopathy | 0.388 | $CD3^+CD4^+CD8^+$ | 165.2 | 148.7 | 6.23 | Clonal | Pre-T LBL |
| G3-50 | 328 | Hepatosplenomegaly | 0.069 (Normal) | $CD3^+CD4^-CD8^-$ | 68.2 | 60.3 | 4.23 | Clonal | Pre-T LBL |

PTCL, Peripheral T-cell lymphoma, not otherwise specified; Pre-T LBL, Precursor T-cell lymphoblastic lymphoma/leukemia.

## Supplementary Table 2. Phenotypic characteristics of the B-cell malignancy developed in 9 *Tet2*$^{-/-}$ mice.

| ID | Age, days | Necropsy findings | Histology | Hematopoietic Disease Phenotype | Blood Count | | | | IgH Rearrangement | Diagnosis |
|---|---|---|---|---|---|---|---|---|---|---|
| | | | | | WBC, K/µL | Ly, K/µL | RBC, M /µL | PTL, K/ µL | | |
| G3-35 | 542 | Hepatosplenomegaly, Lymphadenopathy | Invasion of lympho nodes, intestine, liver | $B220^{lo}IgM^{+}CD19^{+}CD43^{+}TdT^{-}IgD^{+}IgE^{+}IgG1^{-}IgG2a{+}b^{-}Ig\lambda^{-}Ig\kappa^{+}$ | 12.3 | 8.2 | 8.89 | 451 | Clonal | B-ALL |
| G3-38 | 550 | Hepatosplenomegaly, Lymphadenopathy | Invasion of lympho nodes, intestine, liver | $B220^{+}IgM^{+}CD19^{+}CD43^{+}TdT^{-}IgD^{+}IgE^{+}IgG1^{-}IgG2a{+}b^{-}Ig\lambda^{-}Ig\kappa^{+}$ | 11.9 | 7.1 | 5.76 | 249 | N/A | B-ALL |
| G2-8 | 403 | Hepatosplenomegaly, Lymphadenopathy | Invasion of BM, intestine, liver, kidney, lung | $B220^{low}IgM^{+}CD19^{+}CD43^{+}TdT^{-}IgD^{+}IgE^{+}IgG1^{-}IgG2a{+}b^{-}Ig\lambda^{-}Ig\kappa^{+}$ | N.D | | | | N/A | B-ALL |
| G5-87 | 264 | Hepatosplenomegaly, Lymphadenopathy | Invasion of spleen, liver, lympho nodes | $B220^{lo}IgM^{lo/-}CD19^{+}CD43^{+}CD5^{+}TdT^{-}IgD^{+}IgE^{+}IgG1^{-}IgG2a{+}b^{-}Ig\lambda^{-}Ig\kappa^{+}$ | 15.7 | 10.3 | 6.16 | 227 | Clonal | B-ALL |
| G3-322 | 391 | Splenomegaly | Invasion of spleen | $B220^{lo}IgM^{+}CD19^{+}CD43^{+}CD5^{+}TdT^{-}IgD^{+}IgE^{+}IgG1^{-}IgG2a{+}b^{-}Ig\lambda^{-}Ig\kappa^{+}$ | 15.7 | 12.3 | 9.99 | 106 | Clonal | B-ALL |
| G2-25 | 488 | Hepatosplenomegaly, Lymphadenopathy | Invasion of spleen, liver, lympho nodes | $B220^{lo}IgM^{+}CD19^{+}CD43^{+}TdT^{-}IgD^{+}IgE^{+}IgG1^{-}IgG2a{+}b^{-}Ig\lambda^{-}Ig\kappa^{+}$ | 41.9 | 29.8 | 6.01 | 324 | Clonal | B-ALL |
| G3-185 | 272 | Hepatosplenomegaly, Lymphadenopathy | Invasion of spleen, liver, lympho nodes | $B220^{+}IgM^{lo/-}CD19^{+}CD43^{+}TdT^{-}$ | Very High | Very High | 6.45 | 527 | Clonal | B-ALL |
| G5-50 | 358 | Hepatosplenomegaly, Lymphadenopathy | Invasion of spleen, liver, lympho nodes | $B220^{+}IgM^{+}CD19^{+}CD43^{+}CD5^{+}TdT^{-}$ | 183.7 | 172.4 | 6.17 | 412 | N/A | B-ALL |
| G2-7 | 349 | Hepatosplenomegaly, Lymphadenopathy | Invasion of BM, intestine, spleen, liver | $B220^{+}IgM^{+}CD19^{+}CD5^{+}CD43^{+}TdT^{-}$ | 54.1 | 43.7 | 9.37 | 774 | N/A | B-ALL |

B-ALL, acute B-lymphoblastic leukemia.

**Supplementary Table 3. Single cell exome sequencing on selected loci using LK cells from young WT and premalignant *Tet2*$^{-/-}$ mice.**

| Genomic Regions | Mean WT Coverage | Mean Tet2 KO Coverage |
|---|---|---|
| *Flt3* -E14-15 | 5783 | 1707 |
| *Flt3* -E20 | 7575 | 4937 |
| *Mpo* -E6 | 9311 | 7718 |
| *Rhoa* -E3 | 6287 | 7141 |
| *Nlrp1b* -E5 | 2351 | 2009 |
| *Pde1c* -E14 | 3658 | 2598 |
| *Skint6* -E13 | 1809 | 2345 |
| *Cep250* -E11 | 2304 | 1967 |
| *Ank3* -E11 | 3780 | 2354 |
| *Myo7a* -E15 | 4504 | 3687 |
| *Tdrd6* -E1 | 1486 | 1201 |
| *Sirpa* -E2 | 2788 | 3401 |
| *Cd72* -E5 | 2678 | 1965 |

**Supplementary Table 4. Patients samples analyzed by either targeted sequencing or whole exome sequencing.**

| Category | WHO classification | N | Cytogenetic Normal/abnormal In % | Age Median in yrs. |
|---|---|---|---|---|
| MDS | Low risk entities* | 166 | | |
| | RAEB1/2 | 83 | | |
| sAML | | 35 | | |
| MDS/MPN | CMML1/2 | 40 | | |
| | MDS/MPN-U**, RARS-t | 13 | | |
| Total | | 327 | 45/55 | 68 |

*RCUD, RCMD, RARS, MDS-U, 5q-syndrome. TruSeq mutiamplicon deep sequencing targeted most commonly mutated genes including all exons of *TET2*. Of these 202 cases were analyzed by paired exome sequencing involving marrow and $CD3^+$ lymphocyte-derived DNA

**Supplementary Table 5. Antibodies used in the study.**

| Antibody Name | Vendor | Catalog Number | Dilution |
|---|---|---|---|
| TET2 | Abcam | ab124297 | 1:500 |
| | Abiocode | R1086-vp | 1:1000 |
| | Abiocode | R1086-vp2 | 1:1000 |
| FLAG | Sigma | F1804 | 1:1000 |
| β-ACTIN | Cell Signaling Technology | 3700 | 1:1000 |

**Supplementary Methods:**

**Cell sorting and clonality analysis**

For $Lin^{-}c\text{-}Kit^{+}$ (LK), $GFP^{+}$, $B220^{+}$ and $CD3^{+}$ cell purification, FACS cell sorting was applied. Genomic DNA was prepared from splenic $B220^{+}$ or $CD3^{+}$ cells of *Tet2*$^{-/-}$ and WT mice. Clonal rearrangement of IgH segments as well as TCRβ and TCRγ chain segments were amplified by PCR using the primers described previously[1,2]. PCR products were separated by electrophoresis through a 1.2% agarose gel and stained with ethidium bromide.

**Tumor transfer assay**

Tumor transfer experiments were performed to evaluate the malignant nature of the abnormally infiltrated T or B lymphocytes in *Tet2*$^{-/-}$ mice. Briefly, $1x10^{6}$ spleen cells from primary *Tet2*$^{-/-}$ or WT mice were transplanted into sublethally irradiated (600 cGy) F1 recipient mice (CD45.1/CD45.2, n=5) through tail veins (Fig. 2a). Recipient mice were sacrificed when they became moribund or six months post injection. Donor cell chimerism in the PB, spleen and BM was examined at the end of the observation period. These mice were also phenotypically analyzed to determine their hematological phenotype and development of T- or B-cell malignancies. None of the mice receiving WT spleen cells developed pathology or gross evidence of disease within six months of transplantation. By contrast, all the animals receiving spleen cells from *Tet2*$^{-/-}$ mice with T- or B-cell malignancy developed diseases with similar characteristics as those observed in their respective primary donor animals, including elevated WBC counts, lymphocytosis, splenomegaly, enlarged lymph nodes and death.

**RNA interference and western blot**

shRNA sequences targeting mouse *Tet2* (5′-GCTTACAGAATGGAGGGATAA-3′ or 5′-CGGGTTCATATTTGAATCCTT-3′) were inserted into the *pLKO.puro.IRES.mCherry* vector. *pLKO.puro*-shmock or *pLKO.puro*-shTET2 vectors were used to transfect the packaging cell line 293T with two helper packaging plasmids *pCD-NL/BH* (*gag-pol*) and *pVSV-G* (*env*). Tissue

culture supernatants from 293T packaging cells transfected with the above plasmids were collected. Viral particles were concentrated by centrifugation and added to the culture medium of NIH3T3 cells. The viral-infected cells were selected with puromycin selection for 72 h, and then used in the experiments stated in the text. The human *TET2* knockdown HeLa and U2OS lines were created by lentivirus (Sigma-Aldrich, St Louis) transfection using puromycin selection according to the manufacturer's instructions. Nuclear extracts were prepared from cells as previously described. Nuclear extracts were then subjected to SDS-PAGE and western blot with indicated antibodies. Antibodies used and the dilutions at which each antibody was used are stated in Supplementary Table 5. Uncropped scans of each blot are shown in supplementary figure 7.

**Constructs**

Human WT *TET2* cDNA was cloned into *pcDNA*3.1 and *pCDF1-IRES-GFP* vectors. The mammalian codon-optimized mouse *Tet2* and human *TET2* cDNA sequences were synthesized by SynBio Corp., and then ligated into *pcDNA*3.1 and *pCDF1-IRES-GFP* vector.

**Cell culture**

MEL (Mouse ErythroLeukemia), HeLa and NIH3T3 cells were obtained from ATCC. Each cell lines were tested be PCR to confirm that they did not have mycoplasma contamination. NIH3T3 cells were grown in Dulbecco's Modified Eagle's Medium (DMEM) supplemented with 10% fetal bovine serum (FBS) and 1% penicillin-streptomycin. MEL cells were maintained in RPMI1640 with L-Glutamine, 10% FBS and 1% penicillin-streptomycin. HeLa cells were grown in Eagle's Minimum Essential Medium with 10% FBS and 1% penicillin-streptomycin.

**Real-time PCR analysis**

Total RNA was isolated and treated with RNase-free DNase to remove contaminating genomic DNA. First-strand cDNA was synthesized. Real-time PCR was performed using Fast SYBR Green master mix. PCR amplifications were performed in triplicate for each gene of interest along with parallel measurements of *Gapdh* or *β-Actin* cDNA (an internal control). To confirm specific amplification of the desired PCR product, melting curves were analyzed and PCR products were

separated on a 3% agarose gel. The primers used for the amplification of each gene are shown in Supplementary Data 8.

**Array-comparative genomic hybridization**

DNA was extracted from *Tet2*$^{-/-}$ tumors and processed for hybridization to a 244K Whole Mouse Genome Chip (G4122A; Agilent Technologies). Arrays were scanned with an Agilent scanner and analyzed with the Agilent Feature Extraction software. After processing with the Genomic Workbench system 5.0, normalized Array-comparative genomic hybridization (aCGH) values were used to calculate the median ratio at each individual chromosome for each tumor. The reference DNA was obtained from the bone marrow of a wild-type C57BL/6 female mouse.

**Bisulfite-seq and TAB-seq**

Genomic DNA (500ng) of WT and *Tet2*$^{-/-}$ LK cells (from six-week-old mice) was treated using a MethylCode™ Bisulfite Conversion Kit (Thermo Fisher) according to the manufacturer's instructions. DNA was eluted into 10 ul of elution buffer for further analyses. Primers were designed to generate amplicons spanning CpG dinucleotides. Primers were fused to a M13F or M13R sequence to facilitate sequencing. Primer sequences are shown in Supplementary Data 8. Both M13F and M13R primers were required to ensure complete coverage in the sequencing trace. Chromas Lite was used to analyze sequence chromatograms.

The TAB-seq Kit (WiseGene) was used to convert DNA. 500 ng DNA was sheared to an average 400 bp using a bath sonicator (Bioruptor), and then treated with beta-glucosyltransferase for 1 h at 37°C. DNA was purified and concentrated using a QIAquick PCR Purification Kit (Qiagen), followed by treatment with 25 ug of the recombinant Tet1-catalytic domain for 1 h at 37°C. 1 µl Proteinase K was added to the reaction, and the DNA was incubated for 1 h at 50°C. DNA was again purified and concentrated using Micro-Bio-Spin Columns (Bio-Rad) and the QIAquick PCR Purification Kit. The DNA was then bisulfite treated and sequenced as described above.

**Single-cell target sequencing**

Single-cell capture was performed using C1 Single-Cell Auto Prep System (Fluidigm) following the manufacturer's instructions. Briefly, both WT and *Tet2*$^{-/-}$ cells were harvested, washed and re-suspended in C1 DNA wash buffer at a concentration of 166-225 cells/ul. This cell suspension was loaded onto a primed IFC Chip for single-cell capture. After capture, the Chip was imaged with a microscope, and capture efficiency was calculated. Lysis buffer, stop buffer, reaction-enzyme mixture and harvest buffer were then loaded onto the Chip for single-cell lysis and whole-genome amplification.

The harvested amplified DNA was used as a template for PCR. Pairs of primers amplified mFlt3-E14, mFlt3-E20, mMpo-E6 and mRhoa-E3 (Supplementary Data 8). PCR was performed using NEBNext® High-Fidelity 2X PCR Master Mix (NEB). PCR products were purified by AMPure beads (Beckman) and quantified using a Qubit 2.0 Fluorometer (Life Tech). All products amplified from the same Chip and primer were mixed with equal amounts for library construction.

The libraries were generated using the NEBNext ChIP-Seq Library Prep Reagent Set for Illumina (NEB) according to the manufacturer's protocol. An Agilent 2100 BioAnalyzer was used to quantify the libraries. 12-pM diluted libraries were used for sequencing. We performed MiSeq sequencing (Illumina) using MiSeq Reagent Kits v2 (500 cycles) (Illumina). Image processing and sequence extraction were done using the standard Illumina Pipeline. The analytic pipeline and exome sequencing were used for data analyses.

**Supplementary References**

1 Kawamoto, H., Ikawa, T., Ohmura, K., Fujimoto, S. & Katsura, Y. T cell progenitors emerge earlier than B cell progenitors in the murine fetal liver. *Immunity* **12**, 441-450 (2000).

2 Sun, Z. *et al.* PTEN C-terminal deletion causes genomic instability and tumor development. *Cell reports* **6**, 844-854, doi:10.1016/j.celrep.2014.01.030 (2014).
